# Mutant *NRAS^Q61^* shares signaling similarities across various cancer types – potential implications for future therapies

**DOI:** 10.18632/oncotarget.2326

**Published:** 2014-08-08

**Authors:** Igor Vujic, Christian Posch, Martina Sanlorenzo, Adam J. Yen, Aaron Tsumura, Andrew Kwong, Valentin Feichtenschlager, Kevin Lai, Douglas V. Arneson, Klemens Rappersberger, Susana M. Ortiz-Urda

**Affiliations:** ^1^ University of California San Francisco, Mt. Zion Cancer Research Center, San Francisco, USA; ^2^ Rudolfstiftung Hospital, Academic Teaching Hospital, Medical University Vienna, Department of Dermatology, Juchgasse, Vienna, Austria; ^3^ Department of Medical Sciences, Section of Dermatology, University of Turin, Italy

**Keywords:** NRAS, combination therapy, lung cancer, neuroblastoma

## Abstract

Oncogenic mutations in the Neuroblastoma Rat Sarcoma oncogene (NRAS) are frequent in melanoma, but are also found in several other cancer types, such as lung cancer, neuroblastoma and colon cancer. We designed our study to analyze changes in NRAS mutant tumor cells derived from malignancies other than melanoma. A variety of small molecule inhibitors as well as their combinations was tested in order to find beneficial inhibitory modalities in *NRAS^Q61^* mutant lung cancer and neuroblastoma cell lines. Signaling changes after incubation with inhibitors were studied and compared to those found in NRAS mutant melanoma.

All cell lines were most sensitive to inhibition in the MAPK pathway with the MEK inhibitor trametinib. MEK/AKT and MEK/CDK4,6 inhibitor combinations did not show any beneficial effects *in vitro*. However, we observed strong synergism combining MEK and PI3K/mTOR inhibitors in all cell lines. Our study provides evidence that NRAS mutant cancers share signaling similarities across different malignancies. We demonstrate that dual pathway inhibition of the MAPK and PI3K/AKT/mTOR pathway synergistically reduces cell viability in NRAS mutant cancers regardless of their tissue origin. Our results suggest that such inhibitor combinations may be a potential treatment option for non-melanoma tumors harboring activating NRAS mutations.

## INTRODUCTION

Rat sarcoma (RAS) genes encode a family of small guanosine triphosphate(GTP)ases: Harvey rat sarcoma viral oncogene homolog (HRAS), Kirsten rat sarcoma viral oncogene homolog (KRAS) and neuroblastoma viral oncogene homolog (NRAS). Under physiological conditions, these proteins are activated by extracellular stimuli and regulate cellular proliferation, differentiation and survival [1-3]. Mutations are predominantly found in codons 12, 13 and 61. They lead to amino acid substitutions in corresponding proteins causing a continuous activation of downstream pathways that leads to cell division, cell growth and suppression of apoptosis (reviewed in ref. [3,4]). Such mutant RAS oncogenes are found in approximately one-third of all human cancers [1-4].

Mutations in v-Raf murine sarcoma viral oncogene homolog B1 (BRAF), NRAS and Kit are among the most prevalent driving aberrations in cutaneous melanoma with 15-20% affecting the NRAS oncogene [5,6]. While targeting of mutant BRAF with small molecule inhibitors such as vemurafenib and dabrafenib showed promising results in BRAF mutant melanoma, there are no therapeutics available which specifically inhibit mutant NRAS. Meanwhile current treatment approaches focus on inhibiting proteins of the NRAS downstream signaling pathways [7]. Trials are testing different drugs and their combinations in NRAS mutant melanoma patients (NCT01781572; NCT01941927).

NRAS mutations are frequent in melanoma, however many other cancer types also harbor mutant, constitutively active NRAS. Table 1 reports on tumor types in which NRAS mutations have been described and summarizes tumor incidence, mortality, survival and the estimated percentage of tumors bearing NRAS mutations [5,6,8-18].

Our group has recently reported *in vitro* and *in vivo* studies testing a variety of small molecule inhibitors and their combinations in NRAS mutant melanoma cell lines [19]. As shown in Table 1, NRAS mutations in non-melanoma tumors have an important clinical impact on the overall population. Our current study aimed to elucidate the effects of different targeted inhibitors and their combinations in non-melanoma NRAS mutant tumor cells. In order to do so we chose established lung carcinoma and neuroblastoma cell lines with known *NRAS^Q61^* mutations. This study provides evidence that NRAS mutant cancer shares signaling similarities across different malignancies, thus opening up potential treatment options comparable to those proven to be effective in NRAS mutant melanoma tumors. Supplementary Figure S1 shows a simplified schematic of NRAS signaling pathways and the inhibitors used in this study.

**Table 1:** Tumor types with known NRAS mutations Different tumor types in which NRAS mutations have been described. The table reports tumor incidence, mortality, survival and the estimated percentage of tumors bearing NRAS mutations

Cancer	Incidence/100,000/Year	Mortality/100,000/Year	5-Year Survival (%)	NRAS-Mutant Tumors (%)	Reference
Melanoma	21.1	2.7	91.3%	13-25%	5-8
Lung Cancer	61.4	49.5	16.6%	0.7-1%	7-9
Neuroblastoma	1.2	4.78	Age<1yr = 90% Age 1-4yr = 68% Age 10-14yr = 66%	0.8%	10,11
Colon Cancer	45	16.4	64.9%	2.2-5.1%	7,8,12-15
Hepatocellular Carcinoma	7.7	5.6	16.1%	1.3%	7,8,16
Acute Myeloid Leukemia	3.7	2.7	60-80%	10.3%	10,17
Thyroid Cancer	12.2	0.5	97.7%	6.2%	7,8,16
Testicular Cancer	5.5	0.2	95.3%	2.9%	16

## RESULTS

### SW 1271, NCI-H2347, SK-N-AS and CHP-212 cells depend on *NRAS^Q61^* signaling for survival

**Figure 1:**
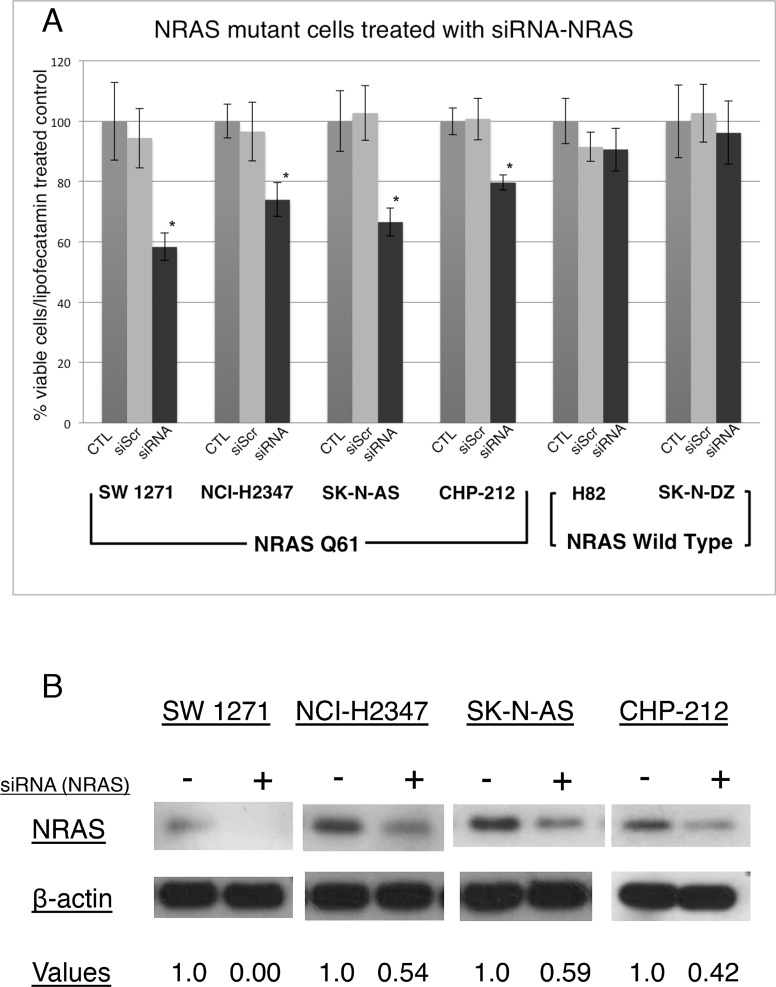
Incubation of *NRAS^Q61^* mutant lung carcinoma and neuroblastoma cells with NRAS siRNA significantly decreases cell viability after 72 hrs of incubation (A)The siRNA directed against the NRAS protein inhibits cell growth in *NRAS*^Q61^ cell lines SW 1271, NCI-H2347, SK-N-AS and CHP-212, but not in *NRAS*^WT^ cell lines NCI-H82 and SK-N-DZ. Neuroblastoma cell lines are more sensitive to NRAS knockdown compared to lung carcinoma tumor cells (n=3, error bars represent the SD of the mean). * *P*<0.5 (Student's T-Test). (B) Immunoblot analyses for *NRAS*^Q61^ mutated cell lines treated with siRNA pool directed against NRAS show a decrease of the NRAS protein. Below the lanes are the densitometry values normalized to a loading control set at 1.0 (ImageJ software).

DNA extraction and the subsequent PCR confirmed the *NRAS* exon 3 Q61 mutations in cell lines SW 1271, NCI-H2347, SK-N-AS and CHP-212. The SK-N-DZ and NCI-H82 cell lines, used as negative controls, did not show mutations in the *NRAS* gene (*NRAS^WT^*). Next we used a commercially available and validated pool of 4 different siRNAs directed against NRAS to perform RNAi-mediated knockdown experiments [20]. As anticipated, the *NRAS^Q61^* mutated lung carcinoma cell lines NCI-H2347 and SW 1271, and the neuroblastoma cell lines SK-N-AS and CHP-212 showed a significant decrease in cell viability after siRNA mediated NRAS knockdown, compared to cells transfected with non-targeting control siRNA and compared to *NRAS^WT^* cells (Figure 1A). The knockdown efficiency of the siRNA pool used was measured and analyzed with the image processing ImageJ software (Figure 1B). Neuroblastoma cells tended to be more sensitive to NRAS knockdown than lung carcinoma cells, still all *NRAS^Q61^* mutated cell lines proved to be dependent on NRAS signaling for cell homeostasis.

### *NRAS^Q61^* mutant lung cancer and neuroblastoma cell lines are most sensitive to inhibition of the MAPK pathway

**Figure 2:**
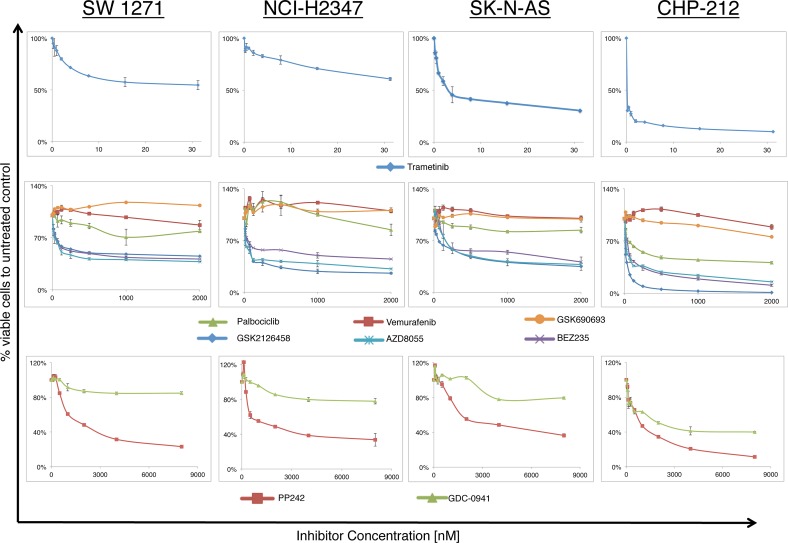
Growth response curves for lung carcinoma and neuroblastoma *NRAS^Q61^* mutant cell lines treated with increasing concentrations of different small molecule inhibitors (n>3, 72hrs incubation) Curves represent the relative change in viability compared to vehicle treated controls. Nanomolar concentrations of the MEK inhibitor trametinib (*Upper panel*) are sufficient to reduce cell viability, whereas inhibitors of the PI3K/AKT/mTOR cascade (PP242, GDC-0941, GSK690693, GSK2126458, AZD8055, BEZ235) and the CDK4,6 inhibitor palbociclib required higher amounts of drug (*Middle and Lower panel*). All cell lines were resistant to the BRAF inhibitor vemurafenib and the AKT inhibitor GSK690693. Only the neuroblastoma cell line CHP212 was sensitive to CDK4,6 inhibition with palbociclib.

In order to evaluate the response of *NRAS^Q61^* mutated lung carcinoma and neuroblastoma cell lines to different small molecule inhibitors, we incubated cells with different inhibitors of the MAPK and PI3K/AKT/mTOR cascade as well as with the CDK4,6 inhibitor palbociclib (CDK4,6i). As expected, all cell lines showed no sensitivity to the specific BRAF^V600^ inhibitor (BRAFi) vemurafenib and to the AKT inhibitor (AKTi) GSK690693 at concentrations used in this study. Two *BRAF^V600^* mutant melanoma cell lines, serving as positive controls to test drug efficiency of the BRAFi and AKTi, showed a marked cell viability decrease after treatment with the same inhibitors (Supplementary Figure S2). In contrast, all *NRAS^Q61^* mutated cell lines were sensitive to the MEK inhibitor (MEKi) trametinib at low nanomolar concentrations, highlighting the importance of MEK signaling in RAS mutant cancer. The *NRAS^WT^* cell lines, which served as negative controls, did not show cell viability decrease following trametinib (Supplementary Figure S3). We also found a decrease in cell viability using the PI3K inhibitor GDC-0941 (PI3Ki), the mTOR1/2 inhibitor AZD8055 (mTOR1/2i), the PI3K/mTOR inhibitors GSK2126458 and BEZ235 (PI3K/mTORi) (Figure 2). Only the neuroblastoma cell line CHP-212 showed a relevant reduction in cell viability in response to treatment with the CDK4,6i palbociclib.

### The combination of MEK and PI3K/mTOR inhibitor synergistically decreases cell viability in *NRAS^Q61^* mutant lung cancer and neuroblastoma cell lines *in vitro*

**Figure 3:**
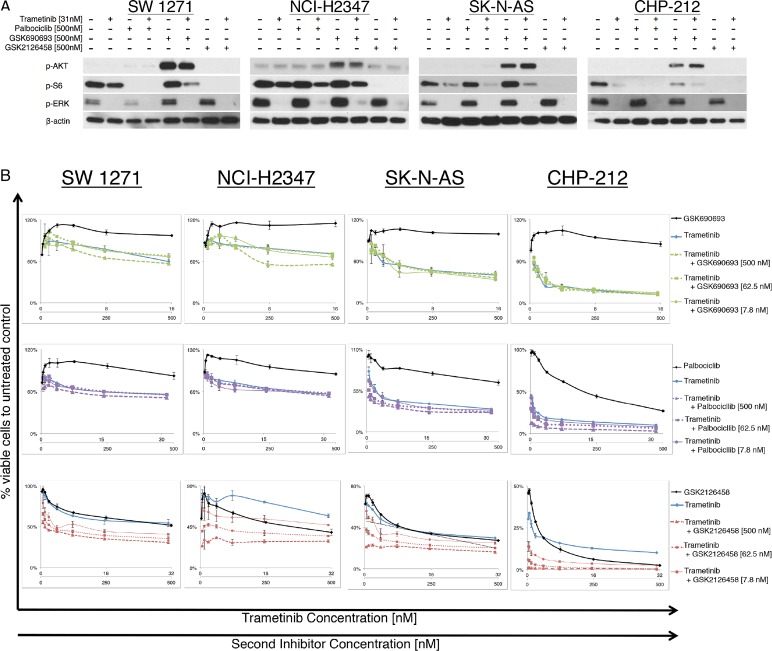
*NRAS^Q61^* lung carcinoma and neuroblastoma cells treated with different inhibitor combinations (A) Immunoblot analyses for downstream effector proteins of the MAPK and PI3K/AKT/mTOR signaling pathways for two lung carcinoma and two neuroblastoma, *NRAS*^Q61^ mutant cell lines, treated with different inhibitor combinations. The combination of the MEK inhibitor trametinib with PI3K/mTORC_1,2_ inhibitor GSK2126458 completely suppressed p-ERK, p-AKT and p-S6 levels in cell lines SW 1271, NCI-H2347, SK-N-AS and CHP-212 (Trametinib = 31nM; GSK690693, palbociclib, PD0332991=500nM) (B) Growth response curves for and two lung carcinoma and two neuroblastoma *NRAS*^Q61^ mutated cell lines (N>3, incubation 72hrs). Cell viability is expressed as the relative number compared to vehicle treated controls. Combinations of the MEK inhibitor trametinib and AKT inhibitor GSK690693 or CDK4,6 inhibitor palbociclib did not result in synergistic effects *in vitro*. Synergistic reduction of cell viability was observed with the combination of a MEK inhibitor and PI3K/mTOR inhibitor.

To test effectiveness of potential combination therapies, we combined the MEKi trametinib with one of the following: AKTi GSK690693, CDK4,6i palbociclib or PI3K/mTORi GSK2126458. Using the Chou-Talalay method to calculate synergism of drug combinations, we observed no beneficial effects when combining MEKi with CDK4,6i or AKTi *in vitro*. In contrast, we observed a strong synergism combining MEKi and PI3K/mTORi in all *NRAS^Q61^* mutated cell lines (Figure 3B). The *NRAS^WT^* negative controls showed a decrease in cell viability following treatment with GSK2126458, but no synergism could be seen when the drug was combined with trametinib (Supplementary Figure S3B)

In this study, we used Cell Titer Glo Assay as a measure of cell viability. However, because Cell Titer Glo Assay functions by detecting metabolically active cells it may have missed live cells with low metabolic activity. We decided to further confirm the induction of apoptosis by combining Annexin V/Propridium Iodide assay with flow cytometry (Supplementary Figure S4). Table 2 reports the GI50 and CI values for all cell lines tested. We now asked the question if the expected signaling changes after inhibitor treatment are comparable to those observed in melanoma cells. Immunoblot analyses for the effector proteins of the targeted pathways showed that only the combination of MEKi and a PI3K/mTORi sufficiently suppressed protein levels of p-ERK, p-AKT and p-S6 in all cell lines, including the *NRAS^WT^* negative controls (Figure 3A and Supplementary Figure S3A). Incubation with the AKTi led to the previously described “paradoxical” AKT hyperphosphorylation in all cell lines tested. This is thought to be due to the occupation of the ATP nucleotide-binding pocket by the AKTi, and is also found in melanoma samples incubated with AKT inhibitors [21-23].

**Table 2:** GI50 and CI and CI r-values for *NRAS^Q61^* mutant lung carcinoma and neuroblastoma cell lines GI50 (Concentration of inhibitors resulting in 50% cell growth inhibition after 72 hours incubation), CI (combination index), and CI r-values for cell lines SW 1271, NCI-H23457, SK- N-AS, and CHP-212. GI50, Cl, and r-value calculations were obtained using Calculsyn Software following the recommendations of Chou-Talalay [19]

Cell line	GI50 Trametinib [nM]	GI50 GSK2126458 [nM]	CI value	*r*
SW 1271	26.9	503	0.09	0.82
NCI-H2347	82	164	0.87	0.95
SK-N-AS	5	63	0.62	0.97
CHP-212	0.5	6.9	0.49	0.99

## DISCUSSION

Oncogenic mutations of the NRAS gene in codons 12, 13 and 61 lead to increased downstream signaling through the well-known NRAS mutant melanoma signaling cascades MAPK and PI3K/AKT/mTOR [24,25]. Whereas, several studies explore the potential use of inhibitors in these pathways in NRAS mutant melanoma, only one study investigated the effects of such therapeutics on NRAS mutated lung cancer cell lines and one study reported on the use of CDK4,6 inhibitors in neuroblastoma cell lines [10,19,24,26–28]. Our study provides evidence that NRAS mutant cancers share similarities in their signaling behavior, which allows the conclusion that MEKi and PI3K/mTORi might also be effective in other malignancies which bear NRAS mutations, including neuroblastoma and lung cancer.

Recent studies have shown that a dual inhibition of MAPK and PI3K/AKT/mTOR pathways leads to a reduction of cell viability *in vitro* and a decrease in tumor size in xenograft models of NRAS mutant melanoma [19,24]. In this study we sought to investigate NRAS mutant lung cancer and neuroblastoma cell lines and showed that certain small molecule inhibitor combinations could also be used in NRAS mutant cancers other than melanoma. While MEKi/AKTi and MEKi/CDK4,6i combinations did not reveal beneficial effects *in vitro*, we were able to prove that MEKi and PI3K/mTORi synergistically reduce cell viability in all cells tested.

Among the different tumors which are known to harbor NRAS mutations (Table 1), we decided to focus our experiments on lung cancer and neuroblastoma cell lines, due to the lack of studies in this specific subset of cells. A study recently reported on the clinical characteristics of NRAS mutant lung cancer, which accounts for approximately 1% of non-small cell lung cancers. The majority of mutations were found in codon 61 (80%). The authors also reported on 6 NRAS mutant lung carcinoma cell lines and their sensitivity to the MEK inhibitors selumetinib and trametinib and their resistance to erlotinib (EGFR-TK inhibitor), crizotinib (ALK/MET/RON/ROS1 inhibitor) and linsitinib (IGF-1R inhibitor) [10]. Even less is known about NRAS mutations in neuroblastoma. A study which reviewed the genetic aberrations of 240 stage IV neuroblastoma patients reported NRAS mutations in 0.83% of the studied tumors [12]. We hypothesized that different cancer types with activating mutations in the same location of NRAS respond to small molecule inhibitors and their combinations in a way comparable to NRAS mutant melanoma cells.

To prove dependency on the mutant NRAS protein, we transiently knocked down NRAS using a commercially available and validated siRNA pool. The knockdown of NRAS decreased NRAS protein expression and cell viability for all four cell lines, thus indicating the need of oncogenic NRAS for cell maintenance. In contrast, the *NRAS^WT^* cell lines did not show a significant decrease of cell viability after NRAS knockdown. Other studies in NRAS mutant lung cancer cell lines support our findings, albeit in different cell lines. Furthermore, our results are in line with NRAS knockdown experiments published previously, proving that NRAS is a *bona fide* oncogene in our subset of cancer cell lines [10, 29].

These findings led us to the assumption that the downstream pathways in our four non-melanoma NRAS mutant cancer cell lines might be comparable to signaling signatures in NRAS mutant melanoma. Thus, we hypothesized that synthetic interference of these pathways might have similar effects on cell viability. The sensitivity profiles to the inhibitors of the two key NRAS downstream signaling cascades MAPK and PI3K/AKT/mTOR were similar to sensitivity profiles found in NRAS mutant melanoma [19]. This indicates the potential use of those inhibitors in NRAS mutant lung cancer and neuroblastoma (Fig 2).

Only the neuroblastoma cell line CHP-212 showed a relevant decrease in cell viability when incubated with the CDK4,6i palbociclib. We interpret this finding as a result of CHP-212 having an *N-Myc* amplification and higher expression of the protein (Supplementary Figure S5). This is in accordance with previously published studies where it was shown that the sensitivity of neuroblastoma cell lines to CDK4,6 inhibitors is correlated with N-Myc expression [28,30,31].

Mounting evidence suggests that dual inhibition of the MAPK and PI3K/AKT/mTOR pathway is capable of achieving growth inhibition in several tumor types *in vitro* and *in vivo* (19,26,32)]. Current clinical trials are testing different inhibitor combinations in solid tumors such as melanoma and lung cancer using therapeutics in these cascades. Whereas MAPK inhibition is believed to be a corner stone of RAS treatment, the rationale behind using these combination regimens is to achieve greater anti-tumoral activity using possible synergisms between different drugs and preventing the development of resistance.

We were able to demonstrate that the combination of a MEKi and a PI3K/mTORi can reduce cell viability in NRAS mutant cancer cell lines other than melanoma. The calculated combination index showed a clear synergistic effect for MEKi and PI3K/mTORi combinations, while it failed to show synergism when combing MEKi with AKTi or CDK4,6i *in vitro*. The inhibition of the MAPK and PI3K/AKT/mTOR pathway was needed to abolish p-ERK and p-S6, two downstream effectors of the NRAS cascade. Even though the addition of AKTi and CDK4,6i was not promising in our study, it is important to mention that other inhibitor combinations, interfering with the above mentioned pathways, might also be effective. We are aware of the fact that a translation of *in vitro* findings into *in vivo* applications bears considerable challenges, as currently available PI3K/mTOR inhibitors are known to have unfavorable side effect profiles. Also, inhibitors that are ineffective *in vitro* might still be beneficial *in vivo*. However, this study adds evidence to the importance of the MAPK and PI3K/mTOR pathway in NRAS mutant cancer and supports that these findings are not limited to NRAS mutant melanoma cells. We also highlight that the rationale for dual pathway inhibition appears to be imperative, as several pathways are activated by mutant NRAS and cross talk between the two pathways exists [26,33,34].

In conclusion, we show that mutant NRAS signaling shares similarities across various malignancies and that NRAS mutant tumors from different tissues can be blocked with same combinations. With the advent of screening for oncogenic mutations in clinical practice, the consideration of different treatment options based on individual mutations has become increasingly important. Our results suggest that inhibitor combinations targeting the MAPK and PI3K/AKT/mTOR pathways downstream of NRAS may be a potential treatment option for non-melanoma tumors harboring activating NRAS mutations.

## METHODS

### Cell culture

The lung carcinoma cell lines SW1271, NCI-H2347 and NCI-H82 and the neuroblastoma cell lines SK-N-AS, CHP212 and SK-N-DZ were purchased from American Type Culture Collection. The *BRAF^V600^* mutant melanoma cell lines SK-MEL-5 and SK-MEL-28 were a generous gift from Boris Bastian. All cell lines were cultured in RPMI-1640 media supplemented with 10% (vol/vol) heat inactivated fetal bovine serum (FBS) and propagated at 37 °C under 5% CO_2_. Cells were passaged and harvested from flasks using 0.05% or 0.25% trypsine-EDTA.

### Mutation analysis

DNA was extracted using the DNeasy Blood & Tissue Kit (Qiagen, Hilden, Germany), according to the manufacturers’ protocol for cultured cells. Touchdown PCR was performed sequentially, using NRAS exon 2 primers (Primer sequence: forward -ACACGTTAAGCTTATTGCATAACTGA; reverse -GGATTTCCATTGCTTAGGCTGAGG) and NRAS exon 3 primers (Primer sequence: forward – GGTTCCAAGTCATTCCCAGTAGCA; reverse - CCAGATAGGCAGAAATGGGCTTG). Sanger sequencing was carried out and the sequences were analyzed with the Mutation Surveyor Version 4.0.9 (Softgenetics, State College, PA, USA)

### siRNA Experiments

For siRNA studies, cell lines were plated in 96 well plates with a density of 4000-8000 cells per well, or 6 well plates with a density of 100000-200000 cells per well. After 24 hours, cells were transfected with the pre-designed ON-TARGET plus Human NRAS siRNA SMARTpool or the ON-TARGET plus Non-targeting Control siRNA (Thermo Fisher Scientific, Waltham, USA) at a final concentration of 25nM, using Lipofectamine 2000 (Invitrogen, CA, USA) as per manufacturer's instructions and incubated for 72h post transfection. All siRNA experiments were performed at least in triplicates.

### Cell Viability Assays

All drugs used in the study were purchased from Selleck Chemicals and ChemieTek and were dissolved in 100% dimethylsulfoxide (DMSO) (Sigma Aldrich, Missouri, USA). Cells were plated in 96-well plates with a density of 4000-8000 cells per well and incubated for 24 h at 37 °C with 5% C0_2_. Then cells were treated with increasing inhibitor concentrations and combinations. Cell viability was measured with the CellTiter-Glo Luminescent Cell Viability Assay (Promega; G7570) according to the manufacturer's protocol. Luminescence was measured on the SynergyHT plate reader (BioTek, Vermont, USA) using Gen5 software (Version 1.11.5). The concentrations of drugs resulting in 50% decrease in cell viability relative to controls (GI50) as well as the combination index (CI) were calculated using CalcuSyn software (Biosoft, Cambridge, UK; Version 2.1). According to the recommendation of Chou-Talalay, a CI <0.9 indicated synergistic effects of drugs; the synergism was further refined as: slight synergism (CI=0.85-0.9), moderate synergism (CI=0.7-0.85), synergism (CI=0.3-0.7), strong synergism (CI=0.1-0.3) and very strong synergism (CI<0.1) [35].

### Apoptosis assays

Cells were plated in 12-well plates and after 24 hour incubation treated with DMSO, trametinib, GSK2126458 or their combinations. After 72hrs apoptosis analysis was performed using the Dead Cell Apoptosis Kit with Annexin V Alexa Fluor 488 & Propidium Iodide according to the manufacturer's protocol (Invitrogen; V13241) with the AccuriC6 Flow Cytometer using the CFlow software (Version 1.0.227.4).

### Immunoblots

Cells were washed with ice cold phosphate buffered saline (PBS), lysed using radio-immunoprecipitation (RIPA) buffer [150mM NaCl, 1% (vol/vol) Nonidet P-40, 0.5% (wt/vol) sodium deoxycholate, 0.1% (wt/vol) SDS] in 50mM Tris HCl (pH8.0) supplemented with protease and phosphatase inhibitors (Pierce; 78442) as described previously (36)]. Protein concentrations were determined using the BCA Protein Assay kit (Pierce, IL, USA; 23225) according to the manufacturer's protocol. Proteins were separated by SDS-PAGE with 4-20% gradient gels (Bio-Rad Laboratories, CA, USA; 456-1096), transferred to an Immobilon-P PVDF membrane (Millipore, MA, USA; IPVH00010), and blocked in 5% dry milk in Tris Buffered Saline, with Tween 20 (TBST) (Sigma-Aldrich). The membrane was incubated with primary and secondary antibodies, and target proteins were detected with ECL detection reagent (Pierce; 32106). β-Actin served as a loading control and was obtained from Sigma-Aldrich. Phosphor-ERK (4370), phosphor-AKT (4060), phosphor-S6 (4857), N-Myc (9405S) antibodies were obtained from Cell Signaling Technology (MA, USA). N-Ras antibody (F155) was obtained from Santa Cruz Biotechnology (CA, USA). The NRAS protein knockdown efficiencies were analysed using the software ImageJ (version 1.49d).

### SUPPLEMENTARY MATERIAL FIGURES


