# Supplementary material for: Mutant NRASQ61 shares signaling similarities across various cancer types – potential implications for future therapies
Source: Oncotarget. 2014 Aug 8;5(17):7936–44. doi: 10.18632/oncotarget.2326 (PMC4202171; doi:10.18632/oncotarget.2326)
Supplement: Supplementary file 1 [file oncotarget-05-7936-s001.pdf]

Mutant *NRAS*^{Q61} shares signaling similarities across various cancer types – potential implications for future therapies

Supplementary Material

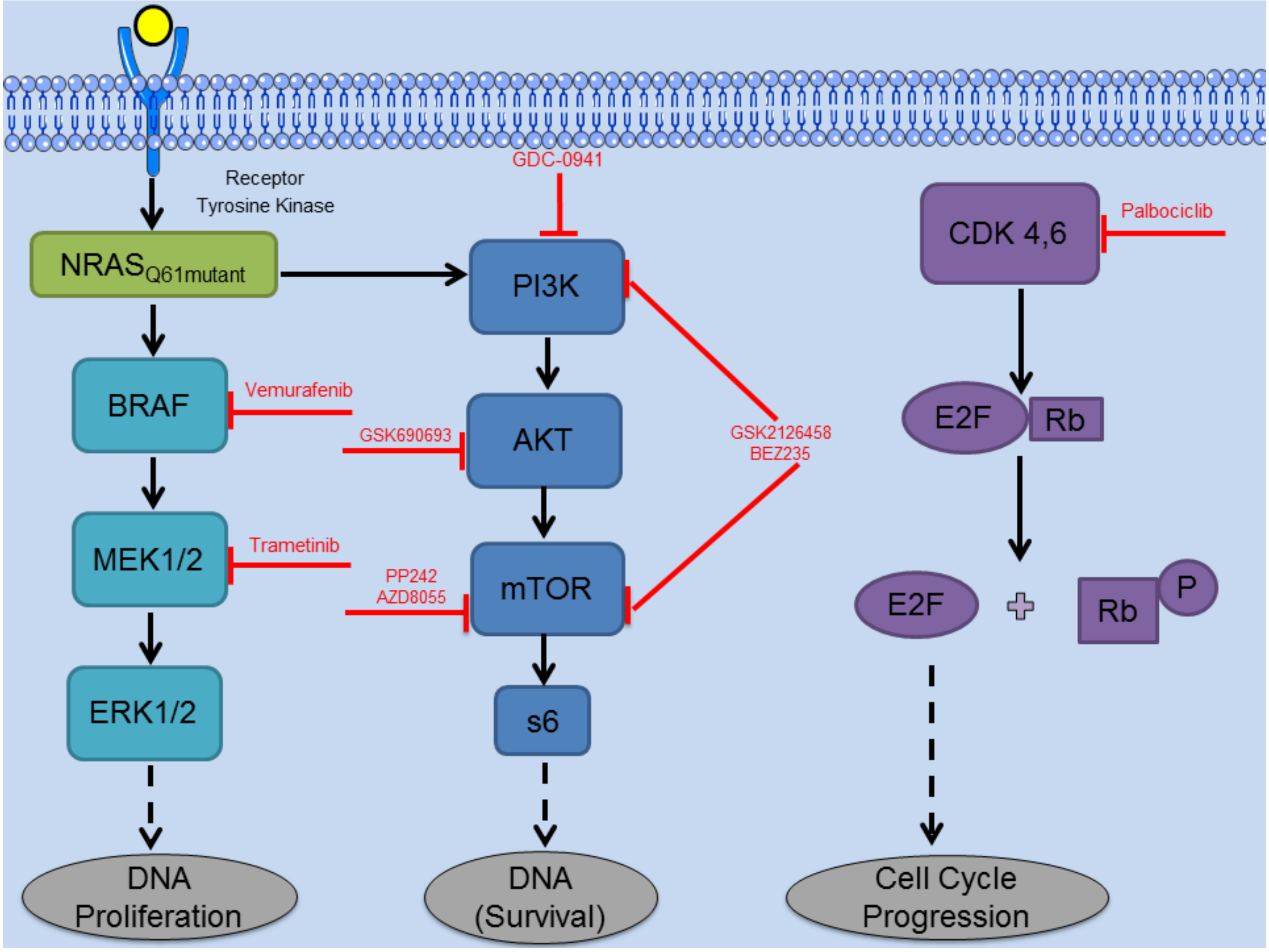

Supplementary Figure S1: Schematic of NRAS signaling pathways. The mutant NRAS protein continuously activates the downstream pathways leading to cell proliferation and enhanced survival. Targeted inhibitors (in red) block different components of the downstreaming cascade.

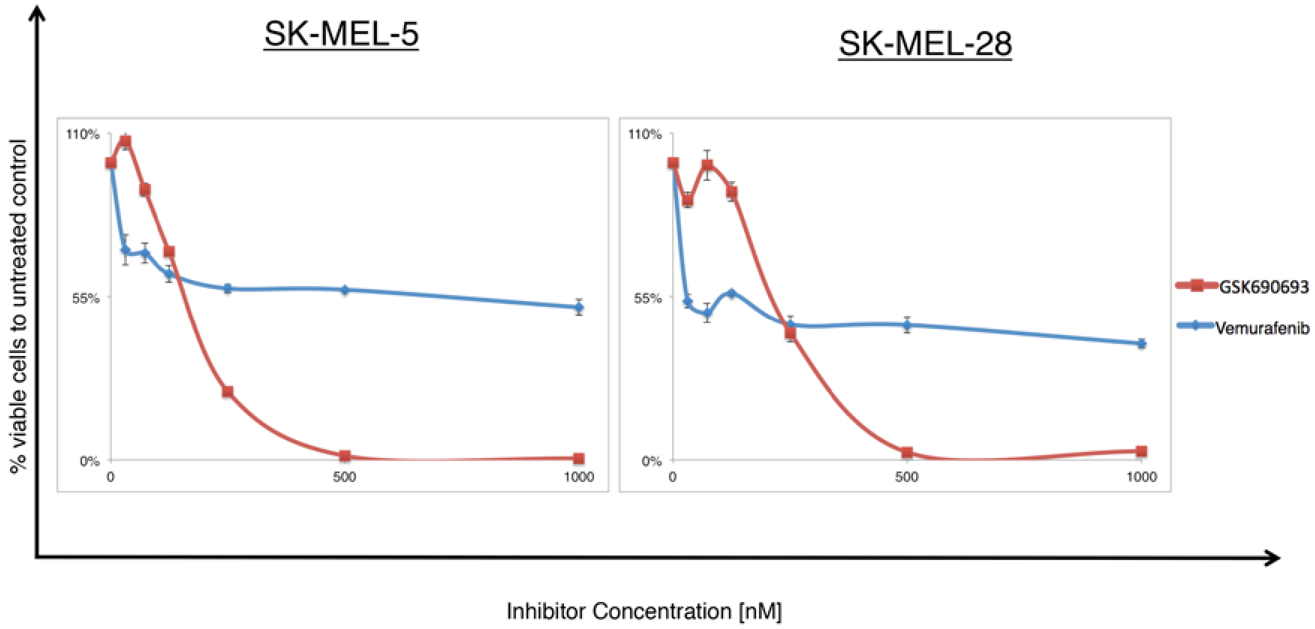

Supplementary Figure S2: Growth response curves for *BRAF*^{V600E} mutant melanoma cell lines treated with vemurafenib and GSK690693. *BRAF*^{V600} mutant melanoma cell lines SK-MEL-5 and SK-MEL-28 show a cell viability decrease relative to vehicle treated controls after treatment with the selective BRAF inhibitor vemurafenib and the AKT inhibitor GSK690693. (N>3, Incubation 72hrs)

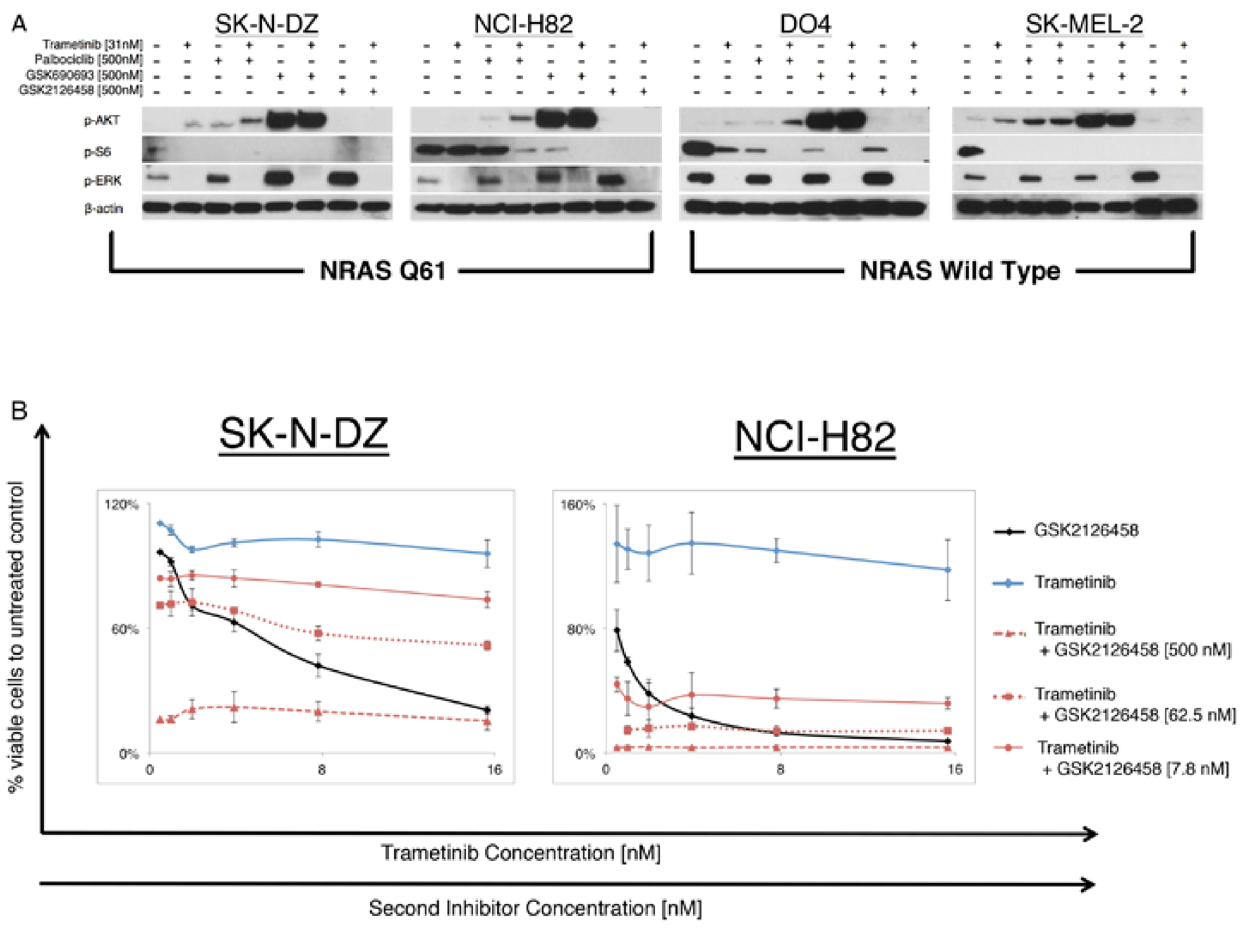

Supplementary Figure S3: Positive (*NRAS*^{Q61}) and negative (*NRAS*^{WT}) controls treated with different inhibitor combinations. (A) Immunoblot analyses for downstream effector proteins of the MAPK and PI3K/AKT/mTOR signaling pathways for *NRAS*^{Q61}mutant melanoma cell lines DO4 and SK-MEL-2 (serving as positive controls), and for *NRAS*^{WT} lung and neuroblastoma cell lines NCI-H82 and SK-N-DZ (serving as negative controls) treated with different inhibitor combinations. The used inhibitors suppress the phosphorylation of the same downstream proteins in all cell lines. (B) Growth response curves for *NRAS*^{WT} lung cancer and neuroblastoma cell lines (N>3, incubation 72hrs). In contrast to *NRAS*^{Q61} mutant cell lines (Figure 3), the cells do not show a decrease in cell viability when treated with the MEK inhibitor trametinib and no synergism when treated with a combination of trametinib and the PI3K/mTOR inhibitor GSK2126458.

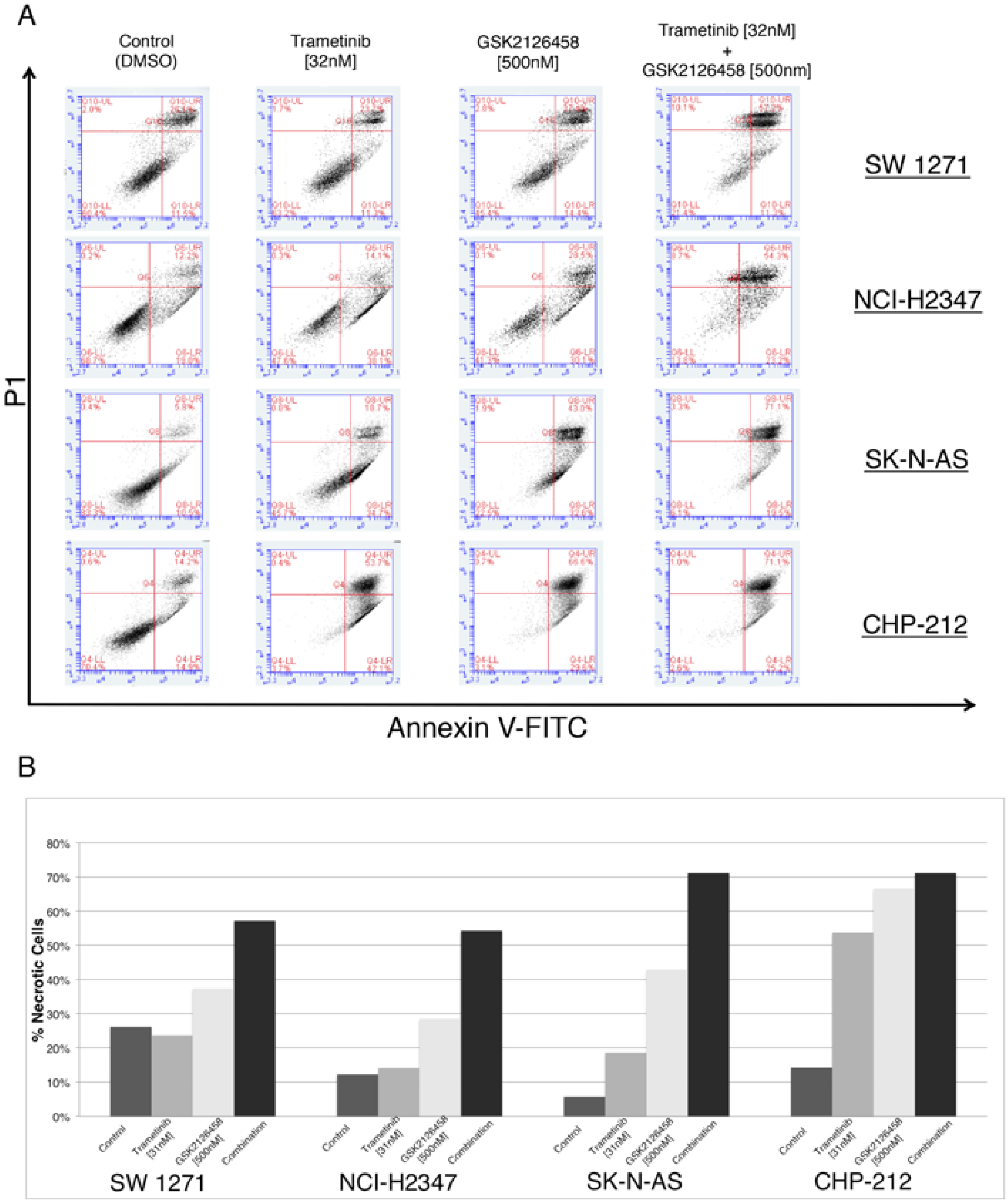

Supplementary Figure S4: The combination of MEK inhibitor and PI3K/mTOR inhibitor leads to decrease in number of viable cells in *NRAS*^{Q61} mutant lung cancer and neuroblastoma cell lines. (A) Representative flow cytometry dot blots from cells treated with different drugs and their combinations as detected by Annexin V-FITC/PI staining. The combination of trametinib and GKS2126458 leads to more cell death then either of the agents alone. (B) Bars represent the relative number of apoptotic cells/condition.

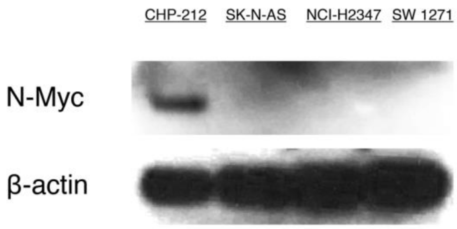

Supplementary Figure S5: Immunoblot analysis of N-myc in *NRAS*^{Q61} lung cancer and neuroblastoma cell lines. Only the neuroblastoma cell line CHP-212 shows a detectable N-myc protein.
